# Supplementary material for: Modulation of Heme-Induced Inflammation Using MicroRNA-Loaded Liposomes: Implications for Hemolytic Disorders Such as Malaria and Sickle Cell Disease
Source: Int J Mol Sci. 2023 Nov 29;24(23):16934. doi: 10.3390/ijms242316934 (PMC10707194; doi:10.3390/ijms242316934)
Supplement: Supplementary file 1 [file ijms-24-16934-s001.zip › ijms-2717144-supplementary.pdf]

Supplementary Table S1. qPCR Primers of the *IL-6R, TLR4, P65/ NFκB, GAPDH. 14-3-3ζ, HMOX1* and *Hemopexin* genes.

IL-6R	Forward	5′-CTC CTG CCA GTT AGC AGT CC-3′	Liu et. al. (2014) [49]
	Reverse	5′-TCT TGC CAG GTG ACA CTG AG-3′	
TLR4	Forward	5′-CAGGATGATGTCTGCCTCGC-3′	Dickson-Copelan et. al. (2015) [47]
	Reverse	5′-TTAGGAACCACCTCCACGCAG-3′	
P65/ NFκB	Forward	5′-TGAACCGAAACTCTGGCAGCTG-3′	Huang et. al. (2010) [50]
	Reverse	5′-CATCAGCTTGCGAAAAGGAGCC-3′	
GAPDH	Forward	5′-TCGGAGTCAACGGATTTGGT-3′	Thakar et. al. (2021) [51]
	Reverse	5′-TTCCCGTTCTCAGCCTTGAC-3′	
14-3-3ζ	Forward	5′-ACCGTTACTTGGCCGAGGTT-3′	Brennan et. al. (2013) [52]
	Reverse	5′-GCAGGCTTTCTCTGGGGAGT-3′	
HMOX1	Forward	5′-GAGTGTAAGGACCCATCGGA-3′	Gueron et. al. (2009) [53]
	Reverse	5′-GCCAGCAACAAAGTGCAAG-3′	
Hemopexin	Forward	5′-AGGAAAAGGCAGCATCCACT-3′	Lawson et. al. (2017) [54]
	Reverse	5′-GTGCCACCTATGCCTTCAGT-3′	
